# The relationship between dispositional empathy, psychological distress, and posttraumatic stress responses among Japanese uniformed disaster workers: a cross-sectional study

**DOI:** 10.1186/s12888-018-1915-4

**Published:** 2018-10-11

**Authors:** Masanori Nagamine, Jun Shigemura, Toshimichi Fujiwara, Fumiko Waki, Masaaki Tanichi, Taku Saito, Hiroyuki Toda, Aihide Yoshino, Kunio Shimizu

**Affiliations:** 1 Division of Behavioral Science, National Defense Medical College Research Institute, 3-2 Namiki, Tokorozawa, Saitama 359-8513 Japan; 2 Department of Psychiatry, School of Medicine, National Defense Medical College, 3-2 Namiki, Tokorozawa, Saitama Japan; 3 Japan Ground Self-Defense Force Medical School, 1-2-24, Ikejiri, Setagaya-ku, Tokyo, Japan

**Keywords:** Disaster workers, Empathy, Interpersonal reactivity index, Identification, Secondary traumatic stress, Posttraumatic stress response, Psychological distress

## Abstract

**Background:**

Disaster workers suffer from psychological distress not only through the direct experience of traumatic situations but also through the indirect process of aiding disaster victims. This distress, called secondary traumatic stress, is linked to dispositional empathy, which is the tendency for individuals to imagine and experience the feelings and experiences of others. However, the association between secondary traumatic stress and dispositional empathy remains understudied.

**Methods:**

To examine the relationship between dispositional empathy and mental health among disaster workers, we collected data from 227 Japan Ground Self-Defense Force personnel who engaged in international disaster relief activities in the Philippines following Typhoon Yolanda in 2013. The Impact of Event Scale-Revised and the Kessler Psychological Distress Scale were used to evaluate posttraumatic stress responses (PTSR) and general psychological distress (GPD), respectively. Dispositional empathy was evaluated through the Interpersonal Reactivity Index, which consists of four subscales: Perspective Taking, Fantasy, Empathic Concern, and Personal Distress. Hierarchial linear regression analyses were performed to identify the variables related to PTSR and GPD.

**Results:**

High PTSR was significantly associated with high *Fantasy* (identification tendency, β = 0.21, *p* < .01), high *Personal Distress* (the self-oriented emotional disposition of empathy, β = 0.18, *p* < .05), and no experience of disaster relief activities (β = 0.15, *p* < .05). High GPD was associated with high *Personal Distress* (β = 0.28, *p* < .001), marital status (married, β = 0.22, *p* < .01), being female (β = 0.18, *p* < .01), medical unit (β = 0.18, *p* < .05), and no experience of disaster relief activities (β = 0.13, *p* < .05).

**Conclusions:**

Among Japanese uniformed disaster workers, high PTSR was associated with two subtypes of dispositional empathy: the self-oriented emotional disposition of empathy and high identification tendency, whereas high GPD was associated with high identification tendency. Educational interventions that aim to mitigate these tendencies might be able to relieve the psychological distress of disaster workers.

## Background

On November 8, 2013, Typhoon Yolanda struck the Philippines and caused large-scale damage: 6300 individuals were killed, 28,688 were injured, and 1062 were missing [1]. The Japanese government responded to a humanitarian assistance request from the Filipino government and sent Japan Self-Defense Forces’ personnel to the affected area. Disaster relief activities consisted of medical assistance, epidemic prevention, and transport of relief supplies [2], which continued until December 18, 2013.

Disasters result in mental health distress not only among survivors but also among disaster workers [3, 4]. These individuals have the burden of rescuing lives in disaster-stricken sites fraught with life-threatening danger. In addition to such direct traumatic stresses, disaster workers can also experience indirect psychological effects of aiding disaster victims, which is defined as secondary traumatic stress [5]. These psychological effects can result in various reactions, including depression and anxiety disorder, in addition to stress-related disorders, such as acute and post-traumatic stress disorder [6]. According to a recent meta-analysis, the pooled prevalence of posttraumatic stress disorder among rescue workers was 10% [7], indicating the magnitude of their work-related traumatic experience on worker’s mental health. Countermeasures for work-related traumatic stress are crucial for disaster workers to prevent adverse mental health outcomes. Coping strategies to mitigate such impacts include sufficient training prior to missions [8–10], awareness of and pride in one’s duties [11, 12], and humor [8, 13, 14]. Minimization of excessive empathy and identification with victims is also recommended to prevent traumatic stress [8, 15–17], which is supported by two empirical studies [15, 17].

Empathy is a multi-dimensional concept with emotional and cognitive components [18]. Davis defined empathy as the “reactions of one individual to the observed experiences of another” and developed the Interpersonal Reactivity Index (IRI), which is a multi-dimensional scale of empathic traits [19]. The IRI consists of four subscales: Perspective Taking (PT; the tendency to spontaneously adopt the psychological point of view of others), Fantasy (FS; the respondents’ tendencies to transpose themselves imaginatively into the feelings and actions of fictitious characters in books, movies, and plays), Empathic Concern (EC; “other-oriented” feelings of sympathy and concern for unfortunate others), and Personal Distress (PD; “self-oriented” feelings of personal anxiety and unease in tense interpersonal settings). Although the construct of self-oriented negative feeling (e.g., PD) is not included in the narrowly defined empathy [20], IRI provides multifaceted information on aspects of dispositional empathy and thus, is one of the most widely used measures to evaluate empathy [21].

As to the relationships between empathy and secondary traumatic stress, Figley reported that individuals who have a great capacity to feel and express empathy tend to be more vulnerable to the traumatic stress in his studies of healthcare workers [22]. Klimecki and Singer suggest that the consequence of empathy take on two paths; one is an “other-oriented,” or compassion that results in prosocial motivation or positive feelings, and the other is a “self-oriented” personal distress that results in withdrawal or the experience of negative feelings [23]. In disaster workers, the latter path might explain their development of posttraumatic stress responses. However, to the best of our knowledge, the relationship between the empathy and secondary traumatic stress has yet to be fully investigated. To expand the theoretical and professional knowledge regarding secondary traumatic stress in the field of traumatic stress, we examined the relationships between dispositional empathy (measured with the IRI) and mental health (posttraumatic stress and psychological distress) among Japan Ground Self-Defense Force (JGSDF) personnel who were deployed on a humanitarian mission in response to the disaster of Typhoon Yolanda in 2013.

## Methods

### Participants and procedure

This survey was conducted as part of the mandatory occupational health management of all JGSDF personnel who were engaged in the humanitarian mission after Typhoon Yolanda (*N* = 283). The self-report survey was administered immediately after they returned home using registered (i.e., non-anonymous) forms. Of all the personnel, only 227 participated and provided written consent (response rate: 80.2%).

### Psychological evaluation

We assessed two outcome measures—posttraumatic stress responses (PTSR) and general psychological distress (GPD)—using the Japanese versions of the Impact of Events Scale-Revised (IES-R) [24] and the Kessler Psychological Distress Scale (K10) [25], respectively. The IES-R is a 22-item self-administered questionnaire (score range: 0–88) that is used to evaluate three domains of PTSR—intrusion, avoidance, and hyperarousal—following a traumatic event [26]. The K10 is a 10-item self-administered questionnaire (score range: 10–50) that was developed by Kessler [27] and is widely used as a tool for evaluating GPD. The originally reported Cronbach’s alpha for internal consistency was 0.91 for the K10 [28] and 0.92 to 0.95 for the IES-R [24]. In this study, Cronbach’s alphas of 0.91 for the IES-R and 0.89 for the K10 were obtained.

Dispositional empathy was evaluated using the IRI [19]. The IRI is a 28-item self-report measure answered on a 5-point Likert scale. It consists of four subscales (PT, FS, EC, and PD), and each subscale contains seven different items (score ranges from 0 to 28). A previous paper reported Cronbach’s alphas of 0.65 for PT, 0.76 for FS, 0.77 for EC, and 0.76 for PD [29]. In this study we obtained Cronbach’s alphas of 0.63 for PT, 0.62 for FS, 0.57 for EC, and 0.65 for PD.

In addition to these psychological measures, the following information was collected based on the previous report by Berger and colleagues [7]: age, sex, marital status, rank, unit (e.g., medical, airlift, others), previous disaster relief experience, exposure to human bodies during relief efforts, and previous psychiatric treatment history.

### Statistical analyses

First, Pearson correlation analyses were performed to explore the correlation between dispositional empathy and PTSR or GPD. Second, analysis of variance was used for the univariate analyses of each personal attribute. Finally, hierarchical linear regression analyses were conducted to investigate factors related to PTSR and GPD. In Step 1, personal attributes including age, sex, marital status, previous deployment experience, psychiatric treatment history were entered as covariates. In Step 2, work-related factors such as rank, unit (e.g., medical or airlift), and exposure to bodies were added as covariates. In Step 3, dispositional empathy evaluated using the four subscales of the IRI (PT, FS, EC, and PD) that were entered as predictor variables.

We used a significance level of *p* < .05. IBM SPSS Statistics for Windows version 22 was used for the statistical tests [30].

## Results

### Personal attributes and the results of the univariate analyses

The average age of the participants was 35.59 ± 7.99 years (mean ± *SD*). As Table 1 demonstrates, those with no previous disaster relief experience scored significantly higher on both PTSR and GPD than those with previous disaster relief experience. In addition, women, officers, and individuals assigned to medical units showed significantly higher scores on GPD than did men, those who were Enlisted/Private, and individuals assigned to “others” units, respectively.

**Table 1:** Personal attributes and the results of the univariate analyses

			PTSR		GPD	
	*N*	%	Mean	*SD*	Mean	*SD*
Sex						
Male	214	94.3	4.6	6.7	12.7	3.6
Female	13	5.7	7.7	6.3	17.2^***^	8.0
Marital status						
Single	81	35.7	4.0	5.9	12.4	3.5
Married	146	64.3	5.2	7.1	13.2	4.4
Rank						
Enlisted/Private	143	63.0	4.3	5.8	12.3	3.1
Officer	84	37.0	5.6	7.9	13.9^**^	5.3
Unit						
Medical	53	23.3	5.9	7.1	14.1^a^	5.3
Airlift	97	42.7	4.8	7.5	13.0	3.8
Others	77	33.9	3.9	5.3	12.0	3.2
Disaster relief experience						
Yes	193	85.0	4.3	6.1	12.6	3.9
No	34	15.0	7.5^*^	8.9	14.9^**^	4.6
Exposure to bodies						
No	216	95.2	4.8	6.8	12.9	4.1
Yes	11	4.8	3.5	4.4	13.5	4.6
Psychiatric treatment history						
No	219	98.2	4.8	6.7	12.9	4.1
Yes	4	1.8	8.5	7.2	11.0	2.0

*N* = 227. *PTSR* Posttraumatic stress response evaluated by the Impact of Event Scale-Revised, *GPD* General psychological distress evaluated by the Kessler Psychological Distress Scale, *SD* standard deviation

^a^Individuals who were assigned to medical units showed significantly higher scores than others (*p* < .05, multiple comparisons with the Bonferroni correction)

^*^p < .05, ^**^ *p* < .01, ^***^ *p* < .001

### Psychological measures and correlation analyses

Table 2 presents the summary statistics for the psychological measures and the results of the Pearson correlation analyses between the measures. IES-R was significantly correlated with two IRI subscales: FS (*r* = .28, *p* < .001) and PD (*r* = .25, *p* < .001). K10 showed a significant correlation with three IRI subscales: PD (*r* = .35, *p* < .001), FS (*r* = .23, *p* < .001), and EC (*r* = .18, *p* < .01). There were significant correlations between the IRI subscales, except for PT and PD.

**Table 2:** Results of the psychological measures and Pearson correlation analyses

	IES-R	K10	IRI_PT	IRI_FS	IRI_EC	IRI_PD	Mean	SD	Median	Range	Cronbach’s alpha
IES-R	–	.59^***^	.07	.28^***^	.05	.25^***^	4.77	6.70	2	0–40	0.91
K10		–	.13	.23^***^	.18^**^	.35^***^	12.91	4.09	11	10–39	0.89
IRI_PT			–	.14^*^	.43^***^	.04	15.90	4.09	16	5–28	0.63
IRI_FS				–	.22^**^	.39^***^	9.67	4.28	9	0–22	0.62
IRI_EC					–	.28^***^	16.89	3.59	17	8–28	0.57
IRI_PD						–	10.31	3.88	10	2–22	0.65

Note*. N* = 227. *IES-R* Impact of Event Scale-Revised, *K10* Kessler Psychological Distress Scale, *IRI* Interpersonal Reactivity Index, *PT* Perspective Taking, *FS* Fantasy, *EC* Empathic Concern, *PD* Personal Distress, *SD* standard deviation

^*^p < .05, ^**^ *p* < .01, ^***^ *p* < .001

### Hierarchical linear regression analyses

The results of the hierarchical linear regression analyses for high PTSR and GPD are shown in Table 3. It is reported that any variance inflation factor (VIF) exceeds 10 and tolerance value lower than 0.10 indicates a potential problem of multicollinearity [31]. In this study, variance inflation factor (VIF) ranged from 1.007 to 1.621 and tolerance ranged from 0.617 to 0.993 throughout the hierarchical linear regression analyses, no multicollinearity was observed. Regarding the correlates of PTSR, significant associations were observed for two IRI subscales: FS (β = 0.21, SE = 0.11, *p* < .01) and PD (β = 0.18, SE = 0.13, *p* < .05). Having no experience of disaster relief was also significantly associated with high PTSR (β = 0.15, SE = 1.3, *p* < .05). For the correlates of high GPD status, significant relations were shown for PD (β = 0.28, SE = 0.07, *p* < .001), marital status (married, β = 0.22, SE = 0.61, *p* < .01), being female (β = 0.18, SE = 1.15, *p* < .01), medical unit (β = 0.18, SE = 0.68, *p* < .05), and no disaster relief experience (β = 0.13, SE = 0.73, *p* < .05). Therefore, the two IRI subscales, FS and PD, showed the largest standardized coefficient β in the multiple linear regression models for PTSR and GPD, respectively.

**Table 3:** Results of the hierarchical multiple linear regression analysis

	PTSR Hierarchial Models			GPD Hierarchial Models		
	Standardized Coefficient β			Standardized Coefficient β		
	Step 1	Step 2	Step 3	Step 1	Step 2	Step 3
Personal Attributes						
Age	0.02	0.02	0.02	−0.05	−0.05	− 0.04
Sex^a^	0.11	0.11	0.07	0.26^***^	0.23^**^	0.18^**^
Marital status^b^	0.11	0.12	0.13	0.19^**^	0.18^*^	0.22^**^
Disaster relief experience^c^	−0.18^**^	− 0.17^*^	−0.15^*^	− 0.19^**^	−0.17^*^	− 0.13^*^
Psychiatric treatment history^c^	0.09	0.09	0.1	−0.05	−0.05	− 0.06
Work-related factors						
Rank^d^		0.03	0.01		0.09	0.04
Medical unit^c^		0.09	0.1		0.18^*^	0.18^*^
Airlift unit^c^		0.10	0.06		0.16^*^	0.12
Exposure to bodies^c^		−0.06	−0.03		0.03	0.06
Dispositional Empathy						
IRI_PT			0.05			0.04
IRI_FS			0.21^**^			0.08
IRI_EC			−0.11			0.03
IRI_PD			0.18^*^			0.28^***^
Statistics						
R^2^	0.057	0.071	0.164	0.13	0.166	0.271
ΔR^2^		0.014	0.093		0.036	0.106
F	2.635^*^	1.815	3.150 ^***^	6.475^***^	4.696^***^	5.984^***^

*N* = 227. *PTSR* Posttraumatic stress response evaluated by the Impact of Events Scale-Revised, *GPD* General psychological distress evaluated by the Kessler Psychological Distress Scale, *IRI* Interpersonal Reactivity Index, *PT* Perspective Taking, *FS* Fantasy, *EC* Empathic Concern, *PD* Personal Distress

^a^Dummy variable was created (male = 0, female = 1). ^b^Dummy variable was created (single = 0, married = 1). ^c^Dummy variables were created (no = 0, yes = 1). ^d^Dummy variables were created (enlisted/private = 0, officer = 1)

^*^p < .05, ^**^ *p* < .01, ^***^ *p* < .001

## Discussion

We examined the associations between dispositional empathy and the mental health status (PTSR and GPD) of JGSDF personnel who were engaged in the international disaster relief activity after Typhoon Yolanda in 2013. High PTSR was significantly related to both high FS and PD. High GPD was significantly related to high PD. To our knowledge, this is the first paper to report a link between psychological distress or posttraumatic stress and dispositional empathy among disaster workers using quantitative empathy measures.

PD reflects emotional dispositional empathy and represents “self-oriented” feelings of personal anxiety and unease in tense interpersonal settings. Previous research that targeted clinical physicians reported that PD was closely related to secondary traumatic stress [32]. Cognitive neuroscience studies have outlined control mechanisms that regulate whether someone’s empathic reactions are self- or other-oriented, indicating the importance of being aware of the distinction between experiences of the self and others [33, 34]. Our study suggests that individuals with high PD—who have a poor ability to distinguish between self- and other-oriented empathic reactions—are prone to suffering from psychological distress when faced with persons experiencing adversity.

FS, which is the tendency to transpose oneself imaginatively into the feelings and actions of fictitious characters, was also extracted as a significant factor related to high PTSR in this study. Ursano and colleagues have shown the importance of “identification with disaster victims” as a mechanism through which relief workers experience secondary trauma [17]. Cetin and colleagues also reported results that support this finding [15] and warned against excessive identification with disaster victims. Considering the conceptual similarity between FS, which is a type of dispositional empathy, and “identification with disaster victims,” our results also support these previous findings.

Previous disaster relief-related literature suggests the importance of maintaining professional distance and avoiding excessive empathy and identification with victims [8, 15, 17, 35]. A similar trend has been observed in the field of clinical medicine; “detached concern”—a purely cognitive understanding of patients’ emotions, while establishing emotional distance to maintain objectivity and limit exposure to negative emotions—has been traditionally recommended for physicians [36]. Given that those with high FS or PD are prone to suffering from psychological distress when faced with distressed individuals, it is likely that maintaining professional or emotional distance, such as “detached concern,” is especially helpful for such emergency relief workers in order to minimize their psychological distress. However, this does not necessarily suggest that a complete absence of empathy is appropriate. Empathy is multifaceted; “other-oriented” empathy provide individuals with a variety of positive effects such as compassion satisfaction [37] and positive clinical outcomes [38]. One study targeted at physicians reported that compassion satisfaction was strongly associated with EC and PT, while compassion fatigue was more closely related to PD [32]. Therefore, an educational intervention that could reinforce other-oriented empathic reactions while inhibiting self-oriented empathic reactions might be effective for mitigating the psychological distress of emergency relief workers. It will be crucial to examine the effectiveness of such educational interventions through in-depth follow-up studies. These programs may specifically target workers with high PD or FS in order to prevent their posttraumatic mental health difficulties.

In this study, those with no disaster relief experience were identified as being at risk for both high PTSR and GPD, and women, married individuals, and medical unit were identified as being at risk for high GPD. Previous studies that focused on body handlers also reported that women and inexperienced workers had higher levels of distress than men and experienced workers [39]. Our results are consistent with this previous report. As for marital status, the previous literature has reported that unmarried individuals are more prone to suffer from psychological distress than those who are married [40]. Although our study demonstrated the opposite result, we theorize that being married could be not only protective but also risk factor depending on the marital relationship or family situation. For example, for the married personnel who have not built a good marital relationship, the existence of spouse could work as a concern rather than a social support when they are suddenly assigned for disaster relief work. Such background might induce inconsistent outcomes. Since medical staff were more likely to suffer from direct or indirect traumatic stress in such a major disaster, it was reasonable that being a member of medical unit was identified as the risk factor for high GPD.

### Limitations

Our findings should be interpreted within the context of some limitations. First, our study participants were limited to JGSDF personnel and they did not include other defense services; therefore, our results do not represent all JGSDF personnel engaged in disaster relief activities in the Philippines. Second, this study employed a registered (non-anonymous) questionnaire, which has been reported to cause participants to conceal their symptoms, as compared to an anonymous questionnaire [41]. Furthermore, the Japanese sociocultural background strongly stigmatizes the expression of emotional suffering [42]. Thus, there is a strong possibility that participants’ psychological effects were underreported in this study. Third, our cross-sectional study design limits the causal association between the dependent and the independent variables. Finally, this study did not examine other factors that might confound our findings (e.g., pre-deployment mental health status, personal characteristics for coping with stress, previous traumatic experiences, numbers of years of disaster relief experience, compassion satisfaction, and major life event experience). Further studies that include such information are needed to more rigorously identify the psychological effects of these factors for disaster workers.

## Conclusions

Despite some limitations, we demonstrated that PD and FS, which are subscales of the IRI, are related to psychological distress among disaster workers. Situations that increase the risk of secondary trauma are not limited to disaster relief activities but also include humanitarian aid, such as medical and welfare activities. More precise and better understanding of the relationship between dispositional empathy and psychological distress is needed to design better educational interventions to protect the mental health of people who engage in such activities.

## Abbreviations

EC: Empathic Concern
FS: Fantasy
GPD: General psychological distress
IES-R: Impact of Events Scale-Revised
IRI: Interpersonal Reactivity Index
JGSDF: Japan Ground Self-Defense Force
K10: Kessler Psychological Distress Scale
PD: Personal Distress
PT: Perspective Taking
PTSR: Posttraumatic stress response
